# Enhancement of the bone-implant interface by applying a plasma-sprayed titanium coating on nanohydroxyapatite/polyamide66 implants in a rabbit model

**DOI:** 10.1038/s41598-021-99494-4

**Published:** 2021-10-07

**Authors:** Weiyang Zhong, Jianxiao Li, Chenbo Hu, Zhengxue Quan, Dianming Jiang

**Affiliations:** Department of Orthopaedics, The First Affiliated Hospital of Chongqing Medical University, Chongqing, 400016 China

**Keywords:** Orthopaedics, Biomaterials

## Abstract

Solid fusion at the bone-implant interface (BII) is considered one of the indicators of a satisfactory clinical outcome for spine surgery. Although the mechanical and physical properties of nanohydroxyapatite/polyamide66 (n-HA/PA66) offers many advantages, the results of long-term follow-up for BIIs remain limited. This study aimed to improve the BII of n-HA/PA66 by applying plasma-sprayed titanium (PST) and assessing the mechanical and histological properties. After the PST coating was applied to n-HA/PA66 implants, the coating had uneven, porous surfaces. The compression results were not significantly different between the two groups. The micro-CT results demonstrated that at 6 weeks and 12 weeks, the bone volume (BV), BV/tissue volume (TV) and trabecular number (Tb.N) values of the n-HA/PA66-PST group were significantly higher than those of the n-HA/PA66 group. The results of undecalcified bone slicing showed that more new bone appeared to form around n-HA/PA66-PST implant than around n-HA/PA66 implant. The bone-implant contact (BIC) and push-out test results of the n-HA/PA66-PST group were better than those of the n-HA/PA66 group. In conclusion, after PST coating, direct and additional new bone-to-implant bonding could be achieved, improving the BII of n-HA/PA66 implants. The n-HA/PA66-PST implants could be promising for repair purposes.

## Introduction

The clinical use of biomaterials varies by clinicians or surgeons. The definition and understanding of human biomaterials remain to be refined and clarified. A biomaterial implant as an independent unit is designed to make direct interactions with living tissue. The adaptability of implants in vivo is still important. The nano-hydroxyapatite/polyamide66(n-HA/PA66) is a novel biomaterial implant with components of nanohydroxyapatite and polyamide simulating natural bones and has been used clinically in China for more than fifteen years. Many studies, especially preclinical studies^1–4^, have demonstrated that n-HA/PA66 is biocompatible. At the final follow-up in constructing cervical spine stability of the anterior cervical corpectomy decompression and fusion (ACCF), the “radiolucent gap” appeared in the n-HA/PA66 strut and the bone. Our team considered the main reason for the appearance of the “radiolucent gap” to be the insufficient osteogenic induction of n-HA/PA66 itself^2–4^. Making improvements to the bone-implant interface is still a main issue.

At present, surface coating for implants is an important method to improve osteogenesis induction. Our study first used a plasma-sprayed titanium (PST)^4–6^ coating to n-HA/PA66 to improve the bone-implant interface (BII), enhancing osteogenesis induction in a rabbit model and providing additional modification possibilities for biomaterials.

## Methods

### Sample preparation

**Figure 1:**
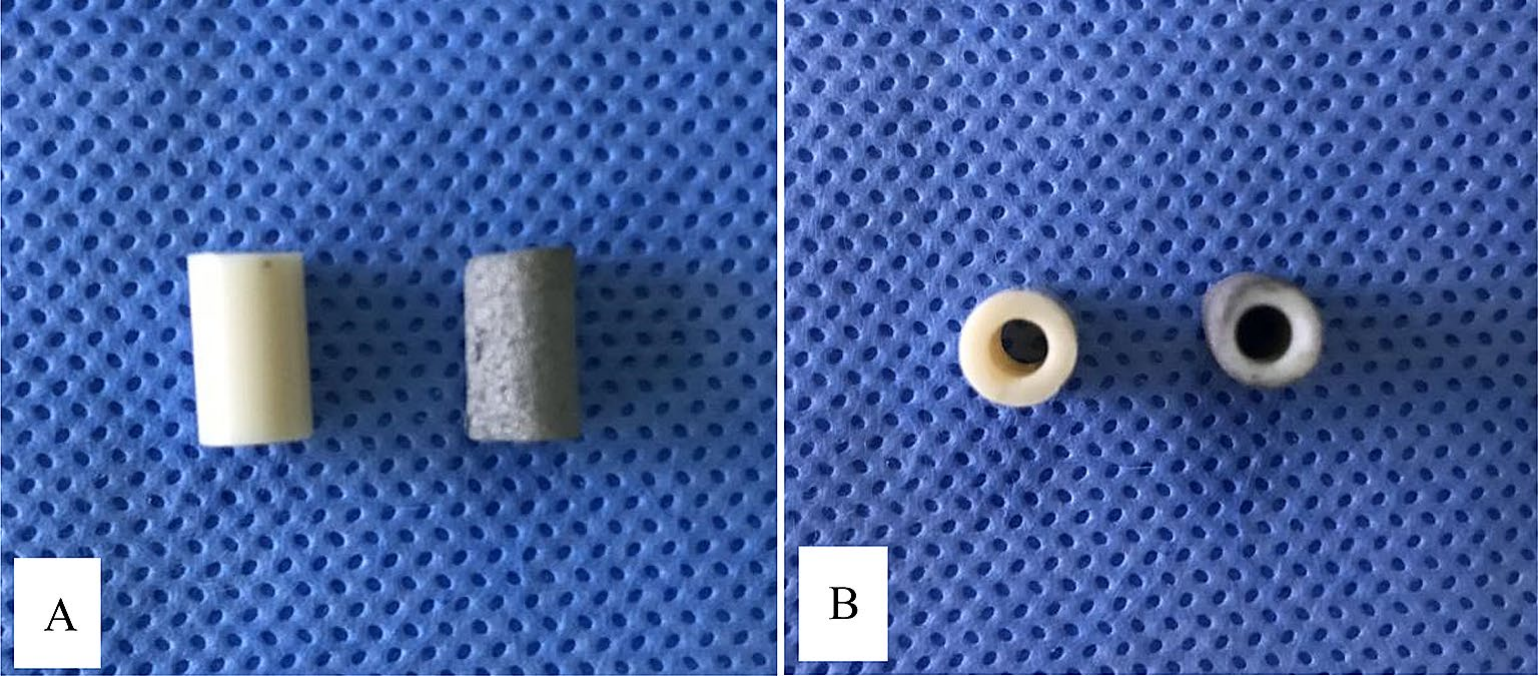
A view of n-HA/PA66 (**A**), n-HA/PA66-PST (**B**) struts.

Cylindrical struts (6 mm*10 mm) of either n-HA/PA66 or n-HA/PA66 with a PST coating were used in the experiment (Fig. 1). The surface features of the n-HA/PA66 samples were qualitatively examined using scanning electron microscopy (SEM). The compression test was performed by an electronic universal testing machine (the loading speed was 0.5 mm/min. Axial pressure was gradually applied until the specimen was destroyed), and the stress–strain curve was recorded. The pull-out tests were performed at 6 w and 12 w, and the rabbits were sacrificed following the intravenous injection of sodium pentobarbital at a dose of 200 mg/kg. After the samples were taken, the specimens were fixed in 4% paraformaldehyde solution, and then the axis was changed to ensure the same axis was used across samples. The axial direction of the implant was adjusted to be consistent with the pushing direction of the universal testing machine, and the pushing speed was adjusted to 0.05 mm/min. The maximum pushing force (Fmax) was recorded, and the results were recorded in the form of the maximum surface shear strength sμ N mm^2^. The formula is sμ = Fmax/S_BIC._ S_BIC_ is the contact area between the implant and bone (S_BIC_ = лDL), where D is the diameter of the specimen and L is the mean thickness of the bilateral cortical bone^7–9^.

### Surgery procedure

**Figure 2:**
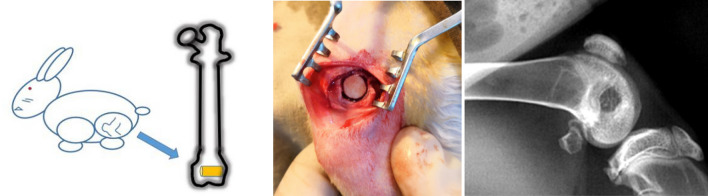
A depiction of the surgery.

The study was carried out in compliance with the ARRIVE guidelines. All methods were carried out in accordance with relevant guidelines and regulations. And all experimental protocols were approved by the Committee of The First Affiliated Hospital of Chongqing Medical University (No: 20187801). Twelve adult male New Zealand white rabbits with a weight of 2.0–3.0 kg were used and were randomly divided into two groups (6 in each group). One femur was selected randomly as the surgical region. General anaesthesia was intravenously administered with 3% sodium pentobarbital solution (1.0 mL/kg) (Sigma-Aldrich Co.). After successful anaesthesia, the surgical site was shaved and disinfected. A longitudinal lateral incision of 3.5 cm was made to expose the femoral groove with medial dislocation of the patella. After flexion of the knee, a bone defect (6 mm width × 10 mm depth) was created in the femoral groove with a bone drill (4 mm width × 2 mm thickness). The n-HA/PA66 and n-HA/PA66-PST struts were implanted at the defect site (Fig. 2). The incision was sutured in layers, and the lateral ligament was sutured tightly to avoid patellar dislocation. Penicillin sodium at 20,000 IU/kg/d (Southwest Pharmaceutical Co., Ltd., Chongqing) was injected intramuscularly 3 days after surgery. The rabbits were kept in separate cages and allowed to move fully after the operation. They were sacrificed after intravenous injection of sodium pentobarbital (200 mg/kg) at 6 and 12 weeks.

### Statistical analysis

Statistical Analysis System software (SAS Institute, Inc., Cary, NC, USA) was applied to analyse the data. Quantitative variables are expressed as the mean ± SD. The chi-squared test was used for the categorical variables. *P* < 0.05 was considered statistically significant.

## Results

**Figure 3:**
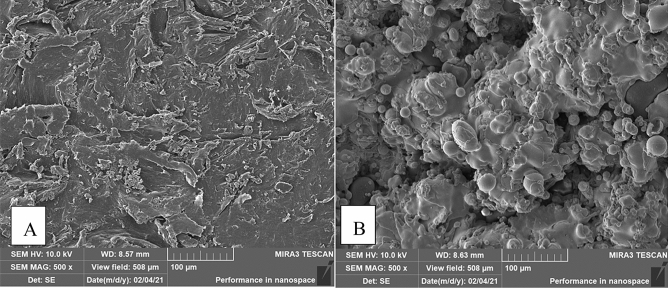
SEM of n-HA/PA66 (**A**) and n-HA/PA66-PST **(B**). The two sample groups were examined using SEM. The surface of the n-HA/PA66 implants was relatively smooth and lacked surface features. The PST coating in on the n-HA/PA66 sample demonstrates a rough topography for bone on growth and cavities for bone ingrowth.

**Figure 4:**
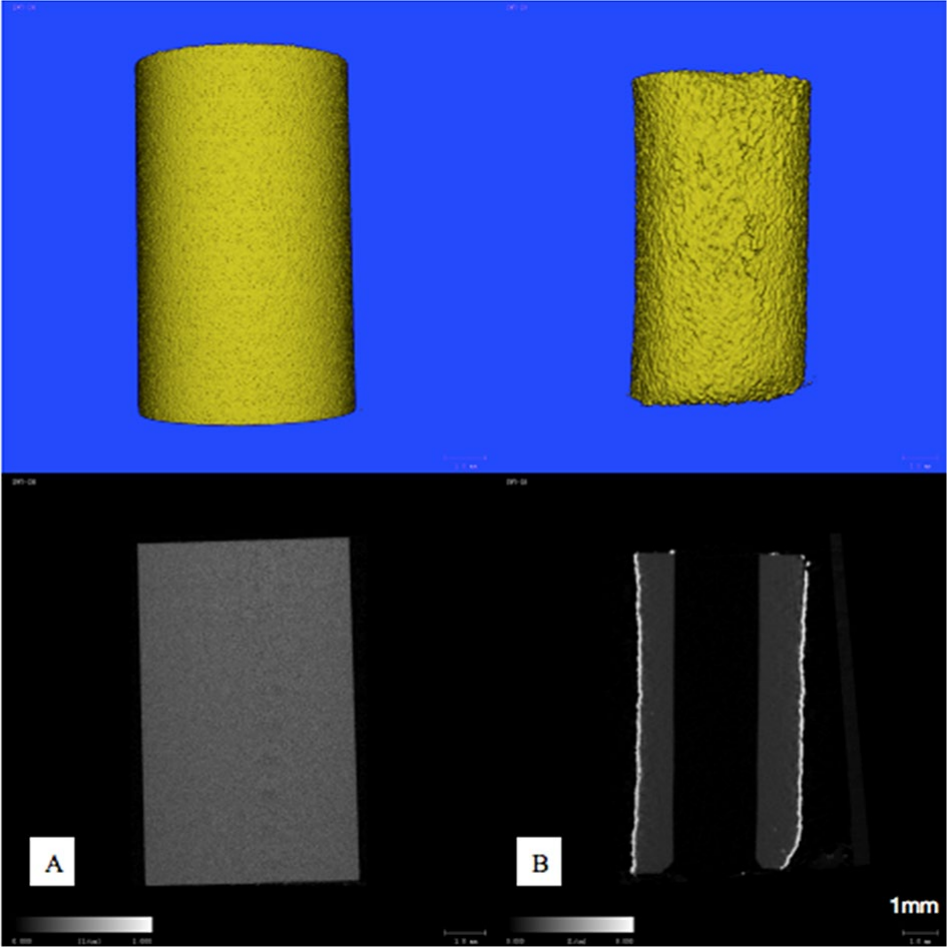
Micro-CT image of n-HA/PA66 (**A**), n-HA/PA66-PST (**B**) implants.

The surface morphology of n-HA/PA66 and n-HA/PA66-PST implants was observed using SEM and micro-CT to confirm the presence of the plasma-sprayed titanium coating on the surface (Figs. 3 and 4). Macroscopic (Figs. 1, 3, and 4) and SEM assessments revealed the lack of surface features on the n-HA/PA66 sample with a relatively smooth interface, whereas the n-HA/PA66 sample demonstrated the presence of the PST layer. The PST coatings all had uneven, porous surfaces that were irregular in appearance in the order of microns, which better promoted osteoblast adhesion and bone growth. The PST layer was uniform along the length of the implant. The plasma-sprayed titanium layer was well integrated with n-HA/PA66 with no macroscopic alterations to the appearance at the titanium-n-HA/PA66 interface.

The rabbits were in good condition after strut implantation. The incision showed varying degrees of swelling, but no infections occurred. Rabbits were sacrificed 6 and 12 weeks postoperatively, and the samples were removed for tests.

**Figure 5:**
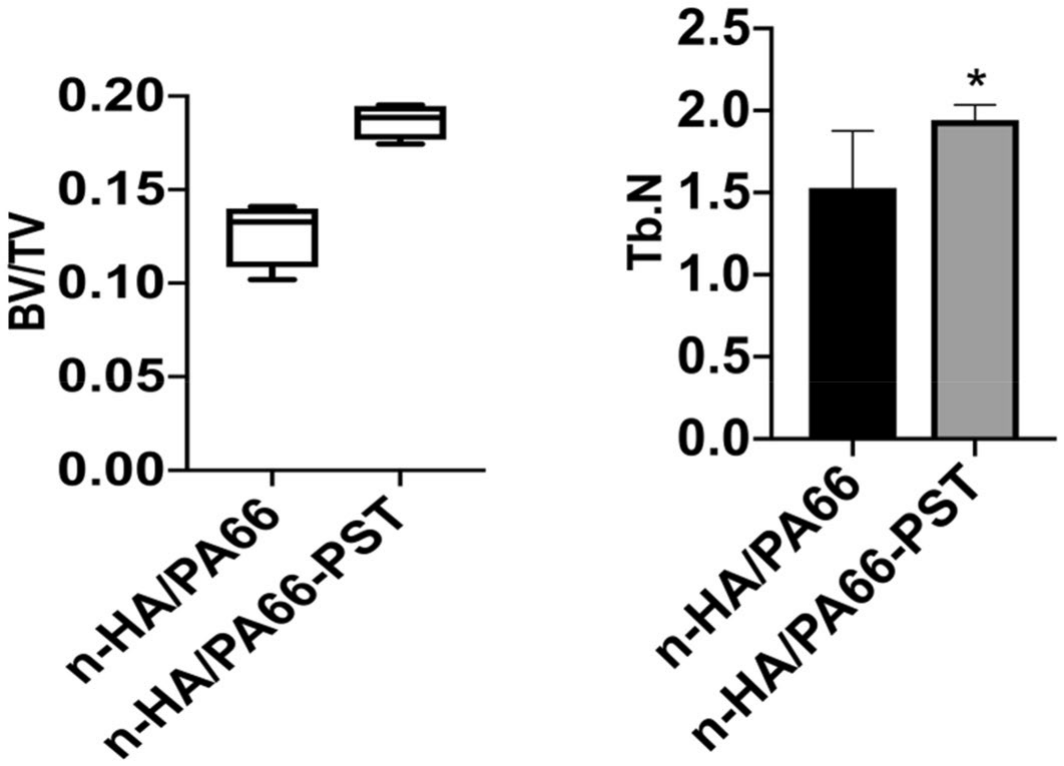
BV/TV and Tb.N at 6 weeks.

**Figure 6:**
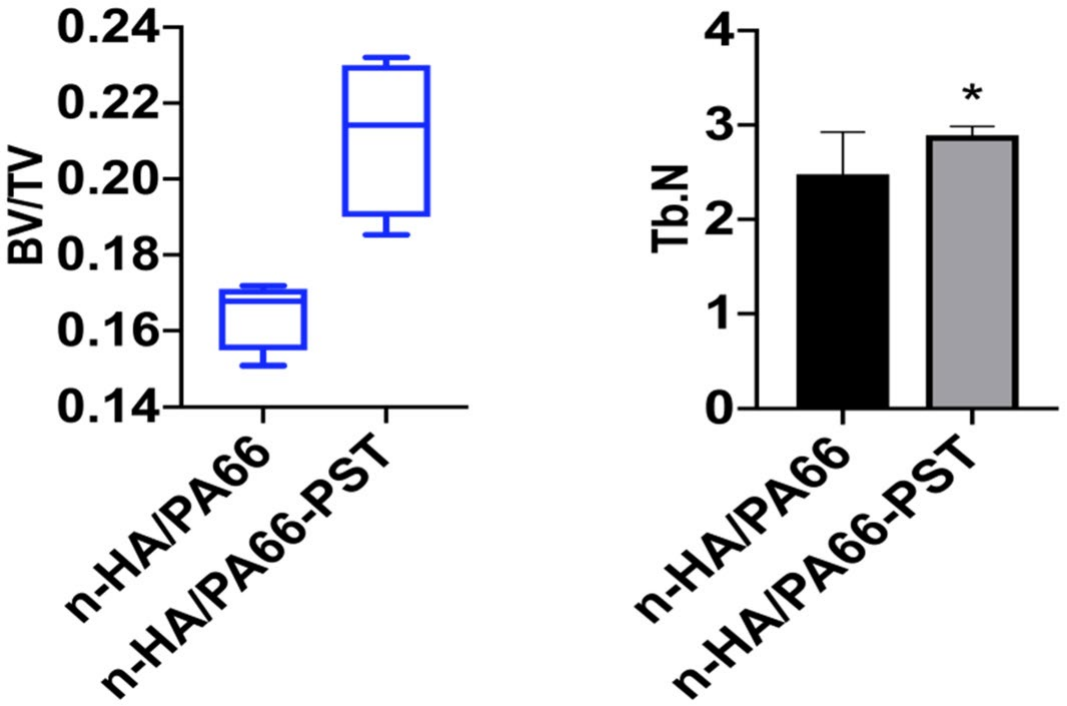
BV/TV and Tb.N at 12 weeks.

**Figure 7:**
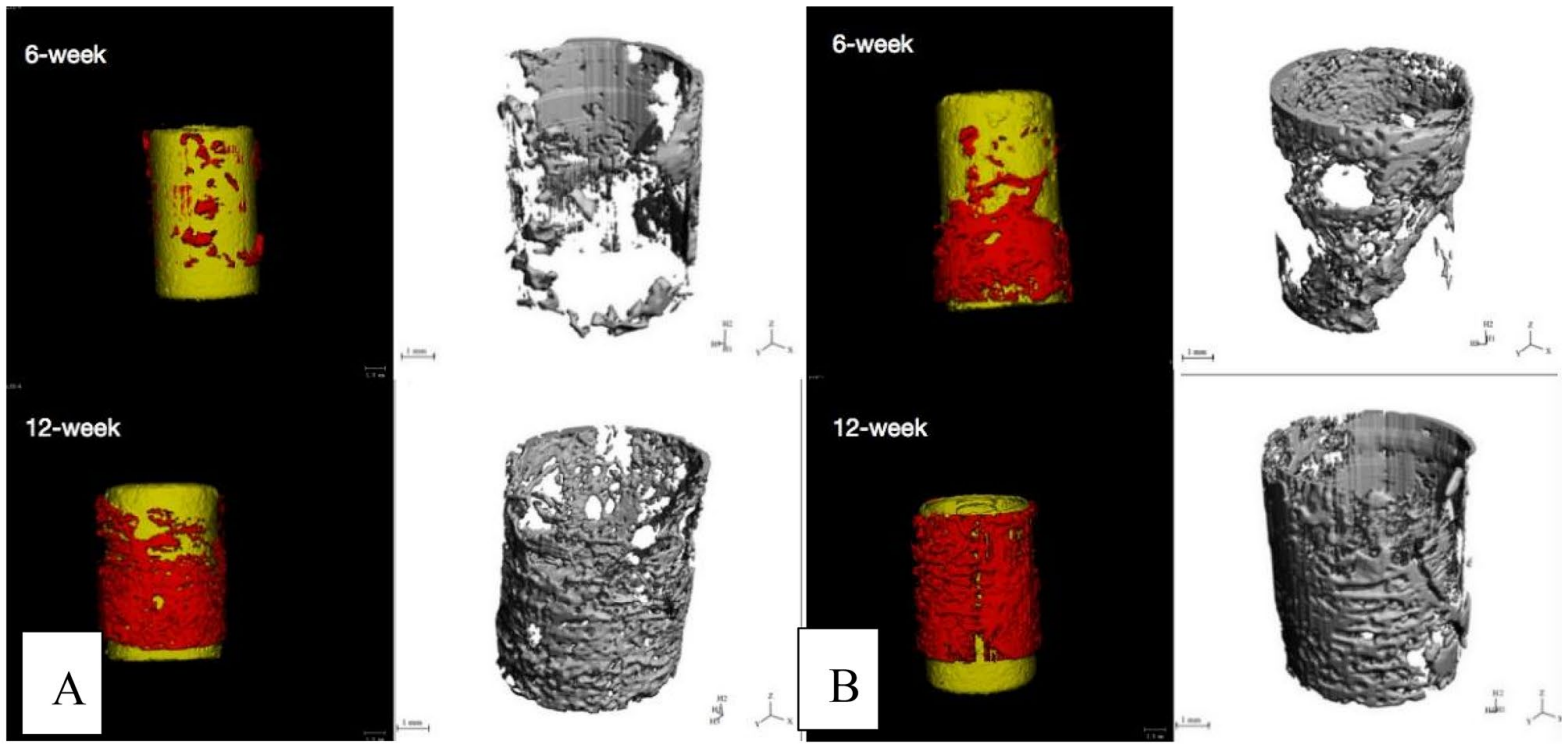
Micro-CT images of n-HA/PA66 (**A**) and n-HA/PA66-PST (**B**) at 6 and 12 weeks.

The three-dimensional micro-CT images showed that more bone tissues were detected around the n-HA/PA66-PST strut than around the n-HA/PA66 strut. At 6 weeks and 12 weeks, the formation of new bone was quantitatively analysed by micro-CT, and the bone volume (BV)/tissue volume (TV) and the trabecular number (Tb.N) were determined. At 6 weeks, the BV/TV values and Tb.N of the n-HA/PA66-PST struts were significantly higher than those of the n-HA/PA66 groups (*P* < 0.05). At 12 weeks, the BV/TV values and Tb.N of n-HA/PA66-PST implants were significantly higher than those of the n-HA/PA66 group (*P* < 0.05) (Figs. 5 and 6). Furthermore, the PST coating on n-HA/PA66 improved the BV/TV and Tb.N comparing those without coating (*P* < 0.05). The three-dimensional micro-CT images showed that more bone tissue was detected within the n-HA/PA66-PST group than in the n-HA/PA66 group (Fig. 7).

**Figure 8:**
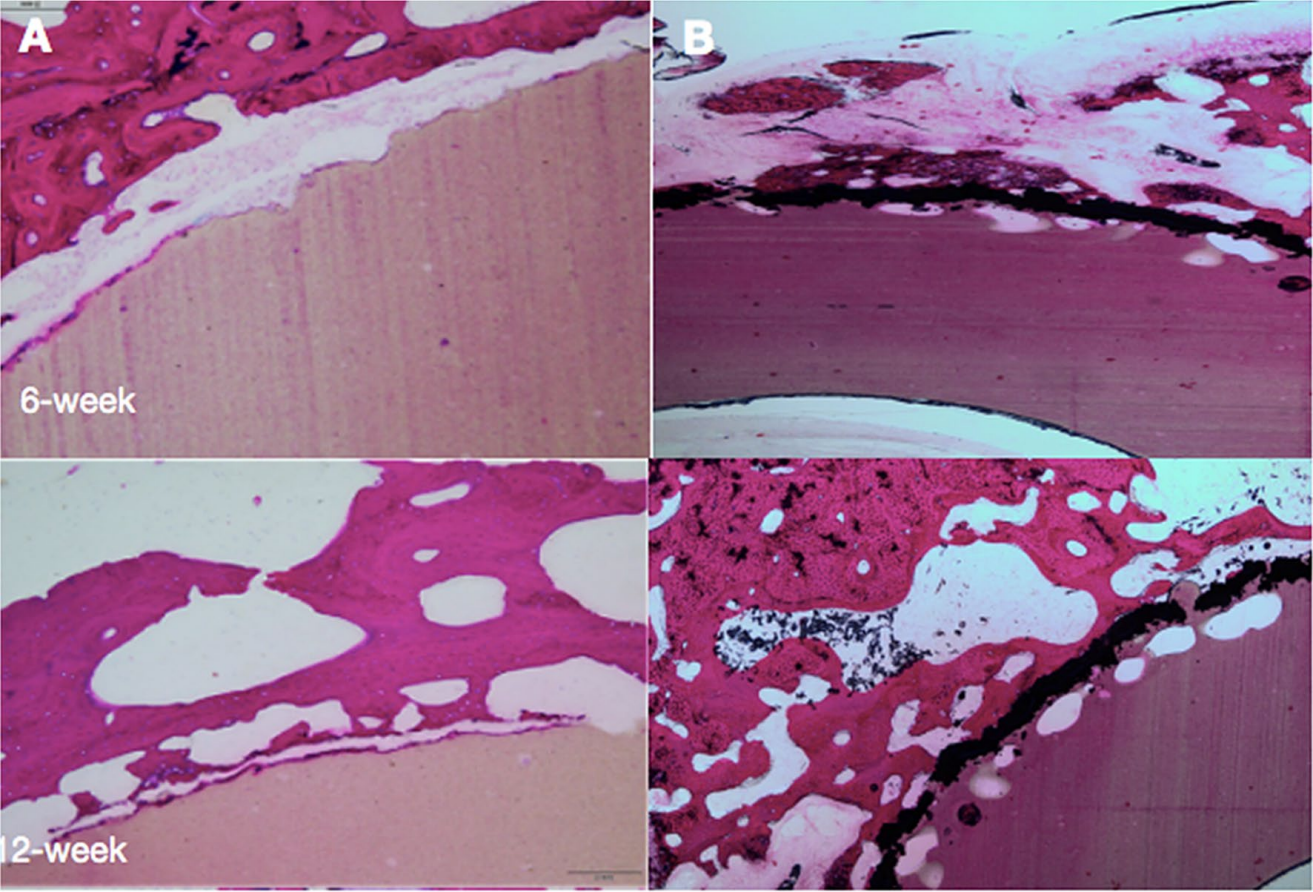
Histological analysis (H&E staining, × 100) of femoral condylar defects and new bone tissue at 6 weeks and 12 weeks after surgery. (**A**) n-HA/PA66; (**B**) n-HA/PA66-PST.

**Figure 9:**
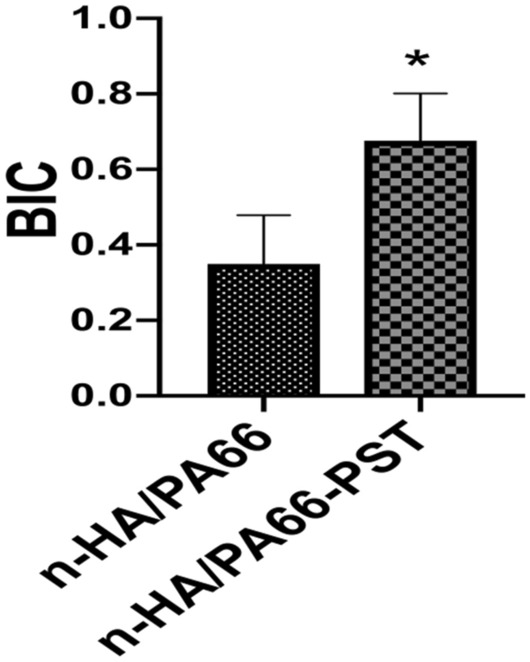
BIC of n-HA/PA66 and n-HA/PA66-PST implants.

At 6 weeks after surgery, n-HA/PA66 and n-HA/PA66-PST were tightly implanted into the surrounding bone tissue and closely integrated. Around the strut of n-HA/PA66-PST, there were more osteoblasts. However, around the strut of n-HA/PA66, few osteoblasts and more fibrochondrocytes were observed (Fig. 8). At 12 weeks, more osteoblasts were observed in the n-HA/PA66-PST group. The osteoblasts did not seem to be in close contact with the strut of n-HA/PA66, but the opposite finding was noted in the n-HA/PA66-PST group. The bone implant contact (BIC) of the n-HA/PA66-PST implants was better (*P* < 0.05) than that of the n-HA/PA66 implants (Fig. 9).

**Figure 10:**
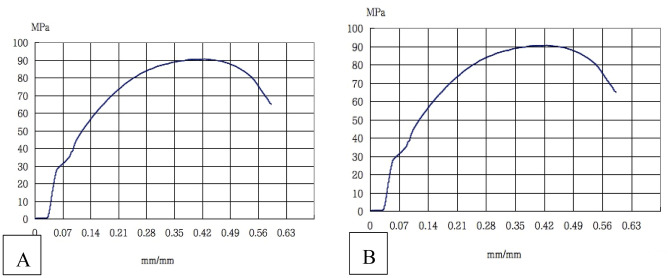
The stress–strain curve of n-HA/PA66 (**A**) and n-HA/PA66-PST (**B**) implants.

The compression results, the compression modulus and compression yield stress, of the two groups are shown in Fig. 10, there was no significant difference (P > 0.05).

**Figure 11:**
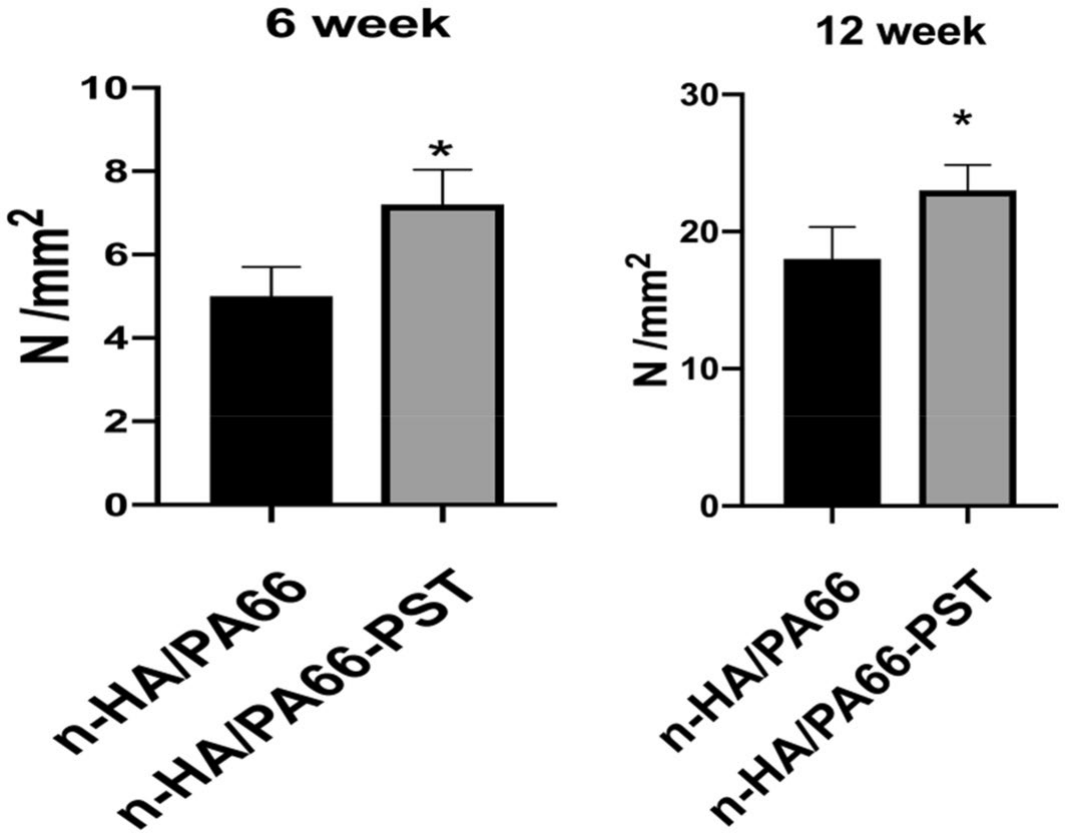
Pull-out test at 6 weeks and 12 weeks of n-HA/PA66 and n-HA/PA66-PST implants.

The results of the two groups at 6 w and 12 w are shown in Table 1 and Fig. 11, with a significant difference (*P* < 0.05), indicating that the osseointegration of n-HA/PA66-PST was better than that of n-HA/PA66.

**Table 1:** The results of the push-out test.

Groups	6 weeks (N/mm^2^)	12 weeks (N/mm^2^)
n-HA/PA66	5.00 ± 0.70	18.00 ± 2.35
n-HA/PA66-PST	7.20 ± 0.84	23.00 ± 1.87
P value	0.0020	0.0058

## Discussion

The ideal bone-implant interface is influenced by surgical, biomechanical, manufacturing, and commercial factors. n-HA/PA66 is a nonmetallic biomaterial implant that is a composite of nanohydroxyapatite and polyamide 66, and it simulates natural bone^1–4^. Our team has reported satisfactory clinical outcomes, but we observed that the “radiolucent gap” was imaged more often between the n-HA/PA66 strut and the bone at both the one-year and final follow-up and that the subsidence rate was higher in patients^2–5^. We discussed this phenomenon, and we considered the reason was insufficient osteogenic induction in the n-HA/PA66 implant. Additionally, a solid BII is vital for treatment success, which remains significantly important and of great research interest. Many methods have been used to modify the surface mechanically or chemically to make the implant adapt to host tissue^4–9^. Biologically, the PST coating on the n-HA/PA66 strut significantly adapted to the bone tissue, providing more new bone growth. Mechanistically, the compression results did not show a significant difference between n-HA/PA66 and n-HA/PA66-PST implants.

In our study, the uneven and porous appearance of PST coating could better promote osteoblast adhesion and bone growth. The three-dimensional micro-CT results showed the BV/TV values and Tb.N of n-HA/PA66-PST implants were significantly higher than those of the n-HA/PA66 group at 12 weeks and the PST coating on n-HA/PA66 improved the BV/TV and Tb.N comparing those without coating. And also at 12 weeks of histological analysis, more osteoblasts were observed in the n-HA/PA66-PST and more osteoblasts were in close contact with the strut of n-HA/PA66-PST.

The study used a well-established rabbit model in which direct new bone ingrowth could be observed^10–13^. The pull-out test performed 6 and 12 weeks after surgery showed that the n-HA/PA66-PST struts achieved better shear strength than the n-HA/PA66 struts. Similarly, according to the BIC results, the BII of n-HA/PA66-PST struts was better than that of n-HA/PA66 struts and supported superior mechanical properties due to increased direct bone contact. The BII findings of gap tissue between n-HA/PA66 implants and host bone tissue that had been reported previously and in our study did not show a significant gap around the new bone tissue and n-HA/PA66-PST implant. The PST coating on the n-HA/PA66 implant provides a means for direct new bone tissue on growth. Although the ideal model provides a means of direct comparison, many factors, such as implants, surgical techniques, and animal mobility, may have complex limitations, affecting the implant response to the host bone tissue. We hope to observe more time points to further determine the long-term benefits of PST coating in bone remodelling, considering more complex biomechanical and biological environments^14–18^.

In conclusion, a PST coating on n-HA/PA66 implants could achieve better fusion in BIIs and improve the osteogenesis-inducing ability of the implant, providing potential surface modification of materials.
